# Implementation of a universal psychosocial screening program across the pediatric cancer treatment trajectory: a standard of care

**DOI:** 10.1007/s00520-026-11317-2

**Published:** 2026-10-17

**Authors:** Lamia P. Barakat, Susanna Vargo, Shannon N. Hammer, Anne Reilly, Charles Phillips, Patricia Burns-Lynch, Lisa A. Schwartz, Megan N. Perez

**Affiliations:** 1 Children’s Hospital of Philadelphia, Philadelphia, PA USA; 2 University of Pennsylvania Perelman School of Medicine, Philadelphia, PA USA

**Keywords:** Childhood cancer, Health equity, Implementation science, Psychosocial screening, Psychosocial Standards of Care, Survivorship

## Abstract

**Purpose:**

Guided by Rapid Evaluation and Assessment Methods (REAM) and using rigorous quality improvement processes, we describe implementation of an evidence-based psychosocial screening program at key transitions in childhood cancer treatment to guide care matched to need.

**Methods:**

Pediatric cancer patients (≥ 11 years) and caregivers (patients 0–17 years) were offered screening questionnaires electronically at 4–6 months, 18–24 months, and 4–6 years post-diagnosis through a phased implementation across three cancer center sites and in their preferred language (English, Spanish, Arabic). Screening questionnaires assessed cancer-related symptoms, health-related quality of life, cognitive function and school needs, caregiver adjustment and family risk, and resource needs. To assess adoption, feasibility, penetration, and sustainability, data related to screening processes, documentation completion rates, and adaptations were tracked and summarized. Qualitative interviews with patients, caregivers, and clinicians, based on the Consolidated Framework for Intervention Research (CFIR), were coded for acceptability and screening program optimization.

**Results:**

Across sites and timepoints, from June 2023 through November 2025, there was a 56% screening completion rate (470/846). Patient physical functioning, emotional functioning, and neurocognitive functioning, caregiver adjustment, and resource needs were consistently flagged for follow-up. Documentation of follow-up varied with rates of 74% for social workers and 34% for medical clinicians. Patients, caregivers, and clinicians endorsed moderate acceptability. Barriers to electronic health record integration complicated clinician workflows. From initiation, over 2 years, 55 adaptations were made to improve workflow, implementation strategies, screening processes, and measures.

**Conclusion:**

We achieved development, implementation, and optimization of a psychosocial screening program across our cancer center.

## Introduction

The diagnosis and treatment of pediatric cancer adversely affect multiple levels of a family’s social ecology, including patient and caregiver physical and psychosocial health [1]. Families with limited instrumental and social resources and preexisting child and family problems are particularly at risk for health disparities and challenges traversing childhood cancer care [2]. As such, there is recognition of the importance of evidence-based psychosocial care for children with cancer and their families for facilitating adjustment through consistent access to and engagement in care matched to needs [3–5]. The 2015 Institute of Medicine Report and the Standards of Psychosocial Care in Childhood Cancer call for improvement in delivery of psychosocial care [1, 3]. Unfortunately, few children’s cancer programs offer care consistent with the standards in a universal, systematic manner, highlighting critical gaps that can limit medical and psychosocial outcomes and magnify health disparities [3, 5].

This project addresses the call to translate the standards into clinical care by focusing on implementing the first standard, youth with cancer and their family members should routinely receive systematic assessments of their psychosocial health care needs [6], and expanding to related standards of neuropsychological assessment (standard 2) [7], assessment of financial needs (standard 5) [8], and psychosocial care for parents (standard 6) [9]. The first standard emphasizes the role of psychosocial screening with patient- and caregiver-reported outcome measures at diagnosis and treatment transitions [6, 10, 11]. Universal screening facilitates early identification of psychosocial risks, initiates preventive interventions to support long-term health-related quality of life, promotes access to targeted, evidence-based care matched to need, reduces implicit bias inherent in nonsystematic assessment, and supports equitable, cost-effective integrated psychosocial services [12, 13]. Further, psychosocial screening at key treatment transitions (e.g., end of active treatment) allows clinicians to assess emerging or persistent patient and caregiver challenges and to adjust care to promote post-treatment adjustment and continued engagement in care [10, 11].

The standards have set expectations for psychosocial care across children’s cancer programs, but implementation has been inconsistent due to barriers at the patient/caregiver, clinician, programmatic, and institutional levels [5, 14]. Facilitators of care consistent with the standards have been identified, and there is value in understanding how to leverage implementation strategies in clinical care to support adoption of screening. Evidence suggests screening is acceptable to patients and caregivers and feasible to implement in clinical care. However, clinicians are concerned with potential challenges in clinic workflow, time and effort required to screen and respond to screening, and costs of meeting identified needs [15]. At the institutional level, it is important to identify screening champions and ensure funding for psychosocial programs that include screening. For example, a recent study suggests few programs view themselves as sufficiently staffed to provide services consistent with the standards, and financial considerations are noted as a barrier to staffing [5]. Also, there was limited growth in psychosocial programs over a 10-year period [5]. Taken together, attention to barriers and facilitators is critical to successful implementation of care consistent with the standards [3].

Building from prior studies in our children’s cancer program [11, 12, 14, 16–18], we designed a universal psychosocial screening program with patient- and caregiver-reported outcome measures at three timepoints in the cancer treatment trajectory. The program was implemented, evaluated, and modified in phases across three Children’s Hospital of Philadelphia (CHOP) Cancer Center disease groups, sites, and screening languages. Implementation strategies were integrated into clinical care, evaluated after each phase, and modified based on ongoing evaluation. Implementation outcomes are described: (1) *adoption* (patient and caregiver completion of surveys), (2) *feasibility* (communication of results to clinicians), (3) *penetration* (clinician documentation of discussion of screening results with patients/caregivers), and (4) modifications for *sustainability* were informed by patient and caregiver acceptability ratings and qualitative feedback from patients, caregivers, and clinicians. We also present *adaptations* or iterative modifications to address identified barriers and promote sustainability of the screening program.

## Methods

**Fig. 1:**
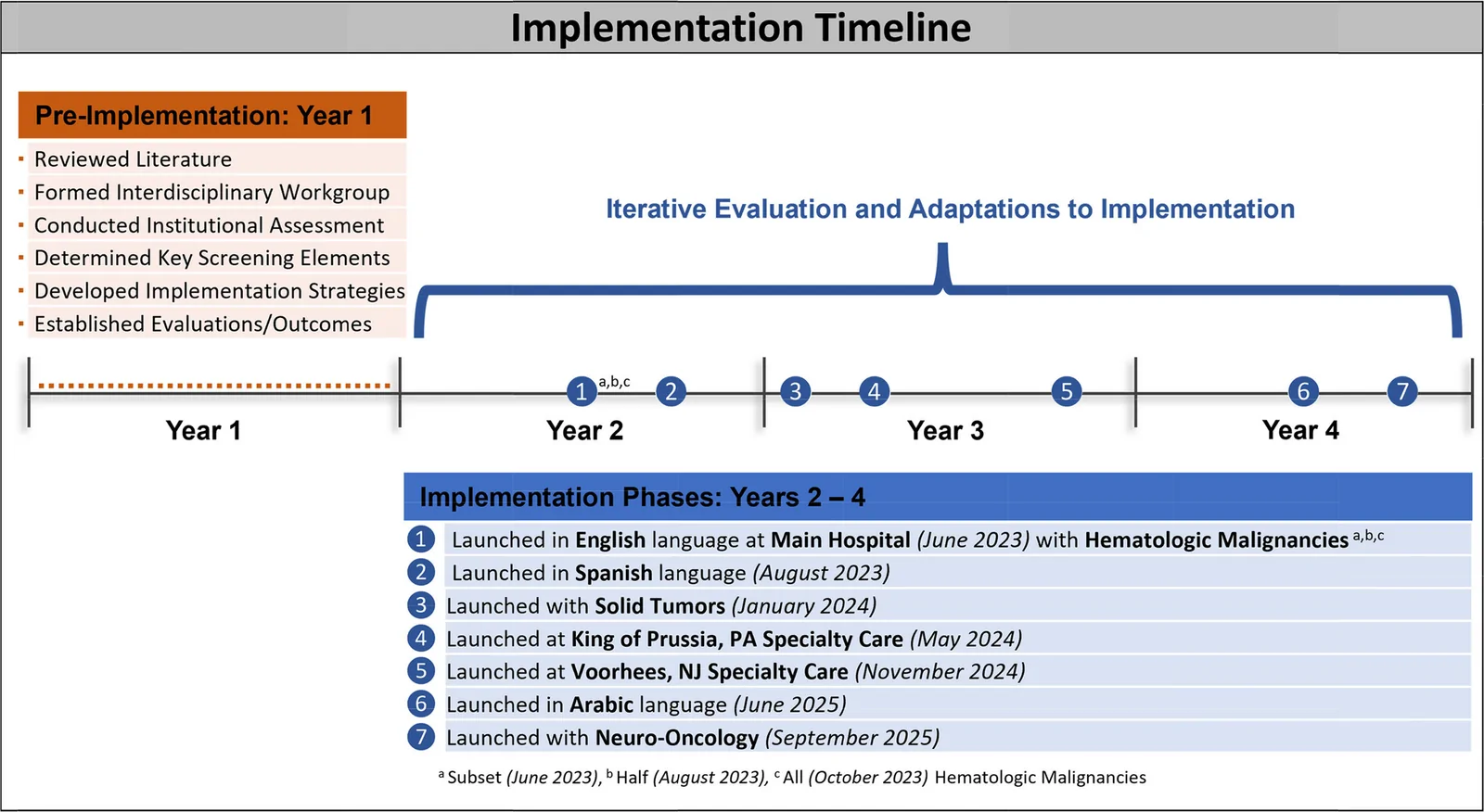
Implementation timeline

This quality improvement project was conducted at the CHOP Cancer Center. We utilized a multiphased approach to implement psychosocial screening (Fig. 1) across its disease groups (hematologic malignancies, solid tumors, brain tumors), sites (main hospital in Philadelphia, PA, and specialty care centers in King of Prussia, PA, and Voorhees, NJ), and most commonly used languages in our center (English, Spanish, Arabic). Institutional review board exempt protocol approval allowed retrospective analysis and dissemination of clinical data and quality improvement processes (CHOP IRB 24–022574; clinical trial number: not applicable).

Rapid Evaluation and Assessment Methods (REAM) [19, 20], guidelines for gathering reliable information on the current services for efficient integration into health care systems and improved responsiveness, patient/caregiver/clinician satisfaction, and service delivery, informed pre-implementation planning and implementation and evaluation. Using REAM [19, 20], iterative modifications and/or enhancements (“adaptations”) to the screening program and its implementation were made based on continuous review of outcomes at each implementation phase and discussion among the screening team, working group members, and relevant clinicians.

### Pre-implementation planning

#### Interdisciplinary workgroup

In late 2021, the screening team formed an interdisciplinary workgroup of CHOP Cancer Center staff (*n* = 10; pediatric psychology, neuropsychology, social work, child life, hospital school program, nursing) and pediatric patient/caregiver community members (*n* = 2) to begin planning a screening program within routine clinical care. The working group appraised the literature review on screening, prepared an institutional assessment of current practice, identified implementation strategies, and provided input throughout planning and preparation.

#### Institutional assessment

Existing screening processes were assessed via REDCap [21] survey of 13 clinicians involved in psychosocial assessments in our cancer program, in accordance with published recommendations for implementing the Psychosocial Standards of Care [22]. Results indicated psychosocial screening was semistructured and primarily occurred immediately following diagnosis or as needed. Evidence-based, psychometrically sound, patient-reported outcome measures were not integrated into screening. Although screening was documented in the electronic health record (EHR), documentation was not templated. We also identified the need for a blueprint to guide the team in systematically matching resources to needs and coordinating multidisciplinary follow-up.

#### Key elements of screening program

Next, core components were determined: timing, patient- and caregiver-reported outcome priorities/domains to impact care (Table 1), and guidance on addressing needs identified through screening (Table 2). A plan for communication with patients, caregivers, and clinicians via the EHR was developed for clinical integration of screening data and documentation of follow-up along with related workflows. Full EHR integration did not allow for comprehensive screening and potential changes in measures, screening processes, and expansion; therefore, we proceeded with screening via REDCap [21] and communication and documentation via the EHR.

**Table 1:** Oncology psychosocial screening program elements

Why?	To enhance clinical care, address health inequities in pediatric cancer, identify areas of risk and resilience, and facilitate timely and targeted assessment/intervention
Who?	*Population*: Patients (≥ 11 years) and caregivers (of patients 0–17 years) in the CHOP Cancer Center *Service*: Hematologic malignancies, solid tumors, brain tumors *Language:* English, Spanish, Arabic
What?	*Screening domains* Cancer-related symptoms Medication management Physical quality of life Social quality of life Emotional functioning Neurocognitive functioning Caregiver adjustment Family factors Resource needs
Where?	*Screening sites* Philadelphia, PA, main hospital King of Prussia, PA, specialty care Voorhees, NJ, specialty care
When?	*Screening timepoints* *Initial diagnosis* (4–6 months post-diagnosis) *Short-term follow-up* (18–24 months post-diagnosis) *Long-term follow-up* (4–6 years post diagnosis/survivorship)
How?	*Screening Process* Screening surveys were delivered to patients and caregivers via a MyChart message with a REDCap [21] link and completed remotely or in clinic. Screening results were shared in an EHR Care Coordination note routed to oncology clinicians and social workers prior to the patient’s clinic appointment.

*CHOP* Children’s Hospital of Philadelphia, *EHR* electronic health record

**Table 2:** Measures with clinical cutoffs indicating needs follow-up

Domain	Measure	Risk score (flagged for follow-up)	Reporter (PT age)			Timepoint
			PT	CGR		
			Self	Proxy	Self	
Cancer-related symptoms	(+) SSPedi^a^	[≥ 1 symptom = 4 or 5]^a^	≥ 11	0–17	**-**	ID, SF
Medication management	PROMIS: Self-Efficacy–Managing Med^b^	*T*-score < 40	≥ 18	-	**-**	ID, SF, LF
Physical quality of life	PedsQL: Physical Functioning^c^	Scale score < 65	-	2–4	**-**	ID, SF, LF
	PROMIS: Physical Function Mobility^d^	*T*-score < 40	11–17	5–17		ID, SF, LF
	PROMIS: Physical Function^e^	*T*-score < 40	≥ 18	-	**-**	ID, SF, LF
	PROMIS: Fatigue^d,e^	*T*-score > 55^c^, 60^d^	≥ 11	5–17	**-**	ID, SF, LF
	PROMIS: Pain Interference^d,e^	*T*-score > 55^c^, 60^d^	≥ 11	5–17	**-**	ID, SF, LF
	(+) PROMIS: Sleep Disturbance^e,f^	*T*-score > 60^e,f^, 59^g^	≥ 11	5–17	**-**	ID, SF, LF
	(−) PROMIS: Physical Activity^g^	*T*-score < 40	≥ 11	5–17	**-**	ID, SF, LF
Social quality of life	PedsQL: Social Functioning^c^	Scale score < 65	-	2–4	**-**	ID, SF, LF
	PROMIS: Peer Relationships^d^	*T*-score < 40	11–17	5–17	**-**	ID, SF, LF
	PROMIS: Social Roles/Activities^e^	*T*-score < 40	≥ 18	**-**	**-**	ID, SF, LF
Emotional functioning	PedsQL: Emotional Functioning^c^	Scale score < 65	-	2–4	**-**	ID, SF, LF
	PROMIS: Anxiety^d,e^	*T*-score > 55^c^, 60^d^	≥ 11	5–17	**-**	ID, SF, LF
	PROMIS: Depressive Symptoms^d^	*T*-score > 55	11–17	5–17	**-**	ID, SF, LF
	PROMIS: Depression^e^	*T*-score > 60	≥ 18	**-**	**-**	ID, SF, LF
	NCCN Distress Thermometer^h^	Rating ≥ 4	≥ 11	0–17		ID, SF, LF
	PAT: Patient Problems^i^	Scale score ≥ 0.50	**-**	0–17	**-**	ID, SF, LF
Neurocognitive functioning	PedsQL: School Functioning^c^	Scale score < 65	-	2–4	**-**	ID, SF, LF
	SCQ^j^	Item 1 = 0 or item 2 = 1^k^	≥ 18	0–17	**-**	ID, SF, LF
	CLDQ: Reading, Math^k^	≥ 2.67, 2.60	**-**	5–17	**-**	ID, SF, LF
	(−) PRISM^l^	≥ 1	**-**	5–17	**-**	ID, SF
	PROMIS: Cognitive Function^m^	*T*-score < 40	≥ 11	8–17	**-**	ID, SF
Caregiver adjustment	PAT: Stress Reaction^i^	Scale score ≥ 0.50	**-**	**-**	0–17	ID, SF, LF
	PAT: Family Beliefs^i^	Scale score ≥ 0.50	**-**	**-**	0–17	ID, SF, LF
	NCCN Distress Thermometer^h^	Rating ≥ 4	**-**	**-**	0–17	ID, SF, LF
Family factors	PAT: Family Problems^i^	Scale score ≥ 0.50	**-**	**-**	0–17	ID, SF, LF
	PAT: Sibling Problems^i^	Scale score ≥ 0.50	**-**	**-**	0–17	ID, SF, LF
Resources	PAT: Social Support^i^	Scale score ≥ 0.50	**-**	**-**	0–17	ID, SF, LF
	PAT: Transit/Money Problems^n^	If ≥ 1 problem endorsed	**-**	**-**	0–17	ID, SF, LF
	NCCN Problem List: Practical Concerns^h^	If ≥ 1 concern endorsed	≥ 18	**-**	**-**	ID, SF, LF

PT (Patient), CGR (Caregiver), ID (initial diagnosis, 4–6 months post-diagnosis), SF (short-term follow-up, 18–24 months post-diagnosis), LF (long-term follow-up, 4–6 years post-diagnosis/survivorship), (+) measure added via adaptation; (-) measure removed via adaptation

Measures:

^a^Symptom screening in pediatrics [flagged for follow-up if ≥ 1 symptom was rated as bothering patient “a lot” (4) or “extremely” (5)] [23]

^b^PROMIS Short Form v1.0–Self-Efficacy for Managing Medications and Treatment 8a [24]

^c^Pediatric Quality of Life Inventory v4.0–Parent Report for Toddlers [ages 2–4] [25]

^d^PROMIS Pediatric-25 Profile v2.0 (patients 11–17); PROMIS Parent Proxy-25 Profile v2.0 (proxy 5–17) [26]

^e^PROMIS-29 Profile v2.1 (patients ≥ 18) [27]

^f^PROMIS Pediatric Short Form v1.0–Sleep Disturbance 4a (patients 11–17); PROMIS Parent Proxy Short Form v1.0–Sleep Disturbance 4a (proxy 5–17) [26, 27]

^g^PROMIS Pediatric Short Form v1.0–Physical Activity 4a (patients ≥ 11); PROMIS Parent Proxy Short Form v1.0–Physical Activity 4a (proxy 5–17) [28]

^h^National Comprehensive Cancer Network v1.2022–Distress Management [29]

^i^Psychosocial Assessment Tool (adapted all-literacy–all ages version, 2019) [16]

^j^School Concern Questionnaire [flagged for follow-up if item 1: “My child is currently meeting their academic potential” = “no” (0) or item 2: “My child needs more educational support to be successful” = “yes” (1)] [30]

^k^Colorado Learning Difficulties Questionnaire [31]

^l^Problem Recognition in Illness Self-Management [32]

^m^PROMIS Pediatric Short Form v1.0–Cognitive Function 7a (patients 11–17) [24]; PROMIS Parent Proxy Short Form v1.0–Cognitive Function 7a (proxy 8–17) [24]; PROMIS Short Form v2.0–Cognitive Function 8a (patients ≥ 18) [26, 27]

^n^Two items (“How will you or other caregivers get to the hospital of clinic?” and “In what areas are there money problems?”) from the PAT: Structure/Resources scale [16]

#### Patient population

Patients aged ≥ 11 years and caregivers of patients 0–17 years were invited to complete surveys via an EHR patient portal message (MyChart) 1 week prior to a scheduled outpatient appointment falling within the relevant screening window. Patients completed self-report measures, and caregivers completed self- and proxy-report measures. Patients were identified via an EHR report based on cancer date of diagnosis and filtered by screening timepoints: 4–6 months post-diagnosis (initial diagnosis), 18–24 months post-diagnosis (short-term follow-up), and 4–6 years post-diagnosis (long-term follow-up) and preferred language (English, Spanish, Arabic, other). With input from our advisory committee and guidance in the First Psychosocial Standard of Care, the three timepoints were selected to coincide with three key moments in cancer treatment and corresponded to times when a structured check-in with families would benefit care. Because of the quality improvement rollout, patients and caregivers could first be offered screening at any timepoint (i.e., surveys were distributed at each timepoint regardless of completion at prior timepoints).

#### Screening measures

Measures were selected based on review of the literature [6], feedback from our interdisciplinary workgroup and community partners about priorities for assessment, consideration of patient/caregiver burden, appropriateness for the age groups, utility in childhood cancer populations, and actionability of response to identified needs (Table 2). For each measure, validation-based cut-points were used to determine results warranting follow-up.

Screening documentati

#### Screening documentation

A templated care coordination note was developed to route screening results to the medical clinician scheduled for the patient’s next appointment and to the social work EHR pool for the patient’s social worker. The note included a score summary table across measures/domains indicating areas requiring follow-up versus no concerns. Separate templated smart phrases for follow-up documentation were developed for the medical and social work teams. Social work documentation also included predefined fields to capture services offered, intervention intensity, and time spent on follow-up.

#### Implementation strategies

Implementation was optimized using evidence-informed strategies catalogued through the Expert Recommendations for Implementing Change (ERIC) project: [33] screening champions; patient, caregiver, and clinician education; minimization of screening burden; and language accessibility. Clinical leaders—medical directors and psychosocial managers—acted as champions by participating in program development, providing feedback, and promoting implementation. Clinician education regarding program purpose, response to screening results (Blueprint for Care, Table 2), and documentation expectations was offered during standing meetings and scheduled sessions and through real-time clinical review and documentation. Standard operating procedures were developed for medical, social work, and screening teams. Patient/caregiver education on the screening program was provided through existing newly diagnosed family education and flyers. To minimize screening burden, surveys were sent 1 week prior to a scheduled clinic visit. Reminders were sent via MyChart and by phone if screening was incomplete within a few days of the appointment. Between June 2023 and November 2024, patients/caregivers without MyChart access or who did not complete screening prior to their appointment were offered portal registration and/or in-clinic completion via iPad; this process was discontinued (see “ Results”). Our hospital’s Language Services translated measures and screening program communications/instructions if translated measures did not exist.

### Implementation evaluation and outcomes

**Fig. 2:**
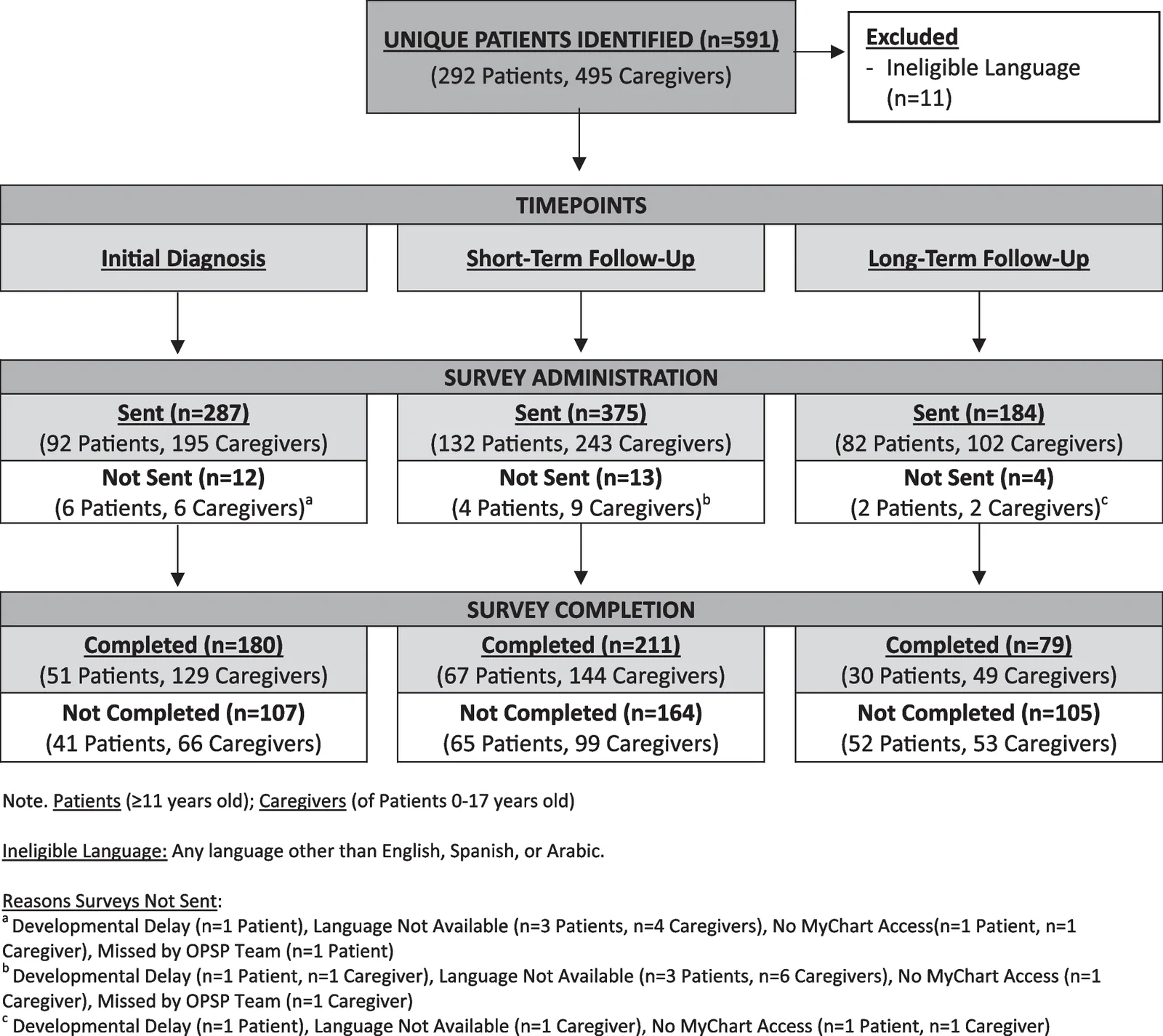
CONSORT

Evaluation was comprised of (1) *adoption*: overall rate of screening completion by timepoint, mode of completion, language, and site; (2) *feasibility*: EHR documentation that results were delivered to clinicians; (3) *penetration*: EHR documentation that patient-caregiver-clinician communication and follow-up occurred as indicated in the medical and social work progress notes; (4) *sustainability*: acceptability ratings from patients and caregivers collected via screening [34] and data on satisfaction through patient, caregiver, and clinician interviews; and (5) *adaptations:* changes made based on review of outcomes, data related to (1)–(4), were continuously tracked (Fig. 2).

#### Qualitative interviews

An interview guide, informed by the Consolidated Framework for Implementation Research (CFIR) [35] and developed in partnership with the working group, evaluated acceptability, screening barriers, impact on care, and implementation strategies among patients and caregivers who completed screening. Approximately 1-month post-screening, with interpreter support as needed, patients and caregivers were invited to participate in a structured phone interview using the interview guide and responses were recorded in REDCap [21]. Clinicians of patients and caregivers who completed screening were emailed a REDCap [21] survey with open-ended questions about workflow integration, impact on patient/caregiver communication, and implementation strategies based on the interview guide. The screening team analyzed patient and caregiver responses inductively by question, with frequencies of perspectives across CFIR domains recorded. Clinician responses were coded as barriers to implementation and effective implementation strategies, with code counts recorded.

#### Adaptations

During implementation, patient, caregiver, and clinician and screening team feedback was regularly reviewed to inform adaptations (i.e., modifications to the program and strategies to address identified barriers). *Adaptations* were recorded in real time in a spreadsheet indicating the modification, date of modification, problem addressed, and its data source.

## Results

Starting in June 2023, the psychosocial screening program rolled out in phases for patient populations, languages, and sites (Fig. 1).

### Adoption, feasibility, and penetration

As of November 2025, 846 surveys were sent to patients (*n* = 306) and caregivers (*n* = 540) across timepoints, with 470 (56%) completed overall (Table 3, Fig. 2), representing 380 unique patients. Nineteen caregivers and four patients completed screening in Spanish and three caregivers in Arabic. As expected, screening was primarily distributed and completed at the main site and with hematologic malignancies (first implementation phase, largest cancer center population).

**Table 3:** Adoption

	Initial diagnosis, *n* (%)		Short-term follow-up, *n* (%)		Long-term follow-up, *n* (%)	
	Patient	Caregiver	Patient	Caregiver	Patient	Caregiver
Surveys completed^a^	51/92 (55%)	129/195 (66%)	67/132 (51%)	144/243 (59%)	30/82 (37%)	49/102 (48%)
Patient age group						
0‒10 years	-	81 (63%)	-	95 (66%)	-	28 (57%)
11‒17 years	40 (78%)	48 (37%)	40 (60%)	49 (34%)	15 (50%)	21 (43%)
≥ 18 years	11 (22%)	-	27 (40%)	-	15 (50%)	-
Mode of completion						
Remotely via MyChart	22 (43%)	77 (60%)	47 (70%)	106 (74%)	22 (73%)	39 (80%)
In clinic	29 (57%)	52 (40%)	20 (30%)	38 (26%)	8 (27%)	10 (20%)
Language						
English	49 (96%)	120 (93%)	66 (99%)	133 (92%)	29 (97%)	47 (96%)
Spanish	2 (4%)	7 (5%)	1 (1%)	10 (7%)	1 (3%)	2 (4%)
Arabic	0 (0%)	2 (2%)	0 (0%)	1 (1%)	0 (0%)	0 (0%)
Site						
Main hospital	44 (86%)	101 (78%)	49 (73%)	109 (76%)	21 (70%)	35 (71%)
KOP specialty care	5 (10%)	21 (16%)	14 (21%)	21 (14%)	3 (10%)	10 (20%)
VNJ specialty care	2 (4%)	7 (5%)	4 (6%)	14 (10%)	6 (20%)	4 (8%)
Service						
Hematologic malignancy	21 (41%)	65 (50%)	40 (60%)	86 (60%)	17 (57%)	31 (63%)
Solid tumors	27 (53%)	55 (43%)	27 (40%)	54 (37%)	12 (40%)	14 (29%)
Brain tumors	3 (6%)	9 (7%)	0 (0%)	4 (3%)	1 (3%)	4 (8%)
Domains flagged for follow-up						
Patient cancer-related symptoms	4/20 (20%)	24/72 (33%)	15/46 (33%)	19/95 (20%)	-	-
Patient medication management	3/10 (30%)	-	5/25 (20%)	-	2/15 (13%)	-
Patient physical quality of life	40/51 (78%)	84/117 (72%)	32/67 (48%)	76/141 (54%)	10/30 (33%)	34/49 (69%)
Patient social quality of life	7/51 (14%)	19/116 (16%)	9/66 (14%)	27/141 (19%)	2/29 (7%)	11/49 (22%)
Patient emotional functioning	25/51 (49%)	81/129 (63%)	28/67 (42%)	52/144 (36%)	9/29 (31%)	17/49 (35%)
Patient neurocognitive functioning	0/1 (0%)	37/82 (45%)	14/65 (22%)	59/121 (49%)	6/28 (21%)	23/46 (50%)
Caregiver adjustment	-	74/128 (58%)	-	53/143 (37%)	-	25/49 (51%)
Family factors	-	18/128 (14%)	-	21/140 (15%)	-	7/47 (15%)
Resource needs	4/10 (40%)	62/128 (48%)	14/25 (56%)	60/141 (43%)	8/15 (53%)	9/48 (19%)

Percentages may not equal 100 due to rounding. Survey measures were completed only by eligible respondents (per validation criteria: age; patient/caregiver); denominators vary by domain

^a^Patient age group, mode of completion, language, site, service, and domains flagged for follow-up are reported for completed surveys only

At initial diagnosis, 55% of patients and 66% of caregivers completed screening, with 43% and 60% via MyChart and 96% and 93% in English, respectively. The most frequently endorsed clinical concerns (flagged for follow-up) for patients were physical quality of life (78%), emotional functioning (49%), and resource needs (40%) and for caregivers were their child’s physical quality of life (72%), emotional functioning (63%) and caregiver adjustment (58%). At short-term follow-up, 51% of patients and 59% of caregivers completed screening, with 70% and 74% via MyChart and 99% and 92% in English, respectively. The most frequently endorsed concerns for patients were physical quality of life (48%), emotional functioning (42%), and resource needs (56%) and for caregivers were their child’s physical quality of life (54%), neurocognitive functioning (49%) and resource needs (43%). At long-term follow-up, 37% of patients and 48% of caregivers completed screening, with 73% and 80% via MyChart and 97% and 96% in English, respectively. The most frequently endorsed concerns for patients were physical quality of life (33%), emotional functioning (31%), and resource needs (53%) and for caregivers were their child’s physical quality of life (69%), neurocognitive functioning (50%), and caregiver adjustment (51%).

Medical clinicians had a documentation rate of 34% (*n* = 129/376) of which 93% (*n* = 120) indicated screening results were discussed with the family and 7% (*n* = 9) indicated results were not discussed. The social work team had a documentation rate of 74% (*n* = 279/376). Among completed notes, 4% (*n* = 10) documented attempted contact but were unable to reach the family, 30% (*n* = 85) indicated no follow-up was needed [of which 78% (*n* = 66) documented professional collaboration], and 4% (*n* = 10) reported the social work team was already engaged with family, and 62% (*n* = 174) documented interventions. Interventions included social work assessment and intervention (89%, *n* = 155), financial assessment and resource referral (33%, *n* = 57), and care coordination and interdisciplinary intervention (23%, *n* = 40). (Percentages reflect the proportion of patients receiving each intervention type and, therefore, do not sum to 100%.) The average duration of interventions was 32.7 (SD = 15.6) minutes, with acuity of 1 (8%), 2 (50%), and 3 (42%), with 3 indicating the highest acuity.

### Acceptability

On the acceptability rating scale (1 = strongly disagree to 5 = strongly agree), average caregiver and patient ratings, respectively, for (1) “Completing the survey prior to or during my clinic visit worked well” (mean = 3.7/5, 3.8/5) and (2) “The amount of time it took to complete the survey was okay” (mean = 3.5/5, 3.8/5) indicated moderate acceptability. Ratings for both items and for caregivers and patients, respectively, were lowest at initial diagnosis [mean(1) = 3.7, 3.8; mean(2) = 3.5, 3.5], higher at short-term follow-up [mean(1) = 3.7, 3.8; mean(2) = 3.6, 3.8], and highest at long-term follow-up [mean(1) = 3.9, 4.1; mean(2) = 3.7, 4.1].

For patient/caregiver interviews, responses to each question fell into contrasting perspectives across CFIR domains, with 90% indicating that the survey was easy to complete, 79% that the length was acceptable, 83% that they understood the screening program and its goals, and 52% that the screening helped them communicate their needs to their health care team (Table 4). Although most (61%) patients/caregivers viewed the cancer center as a resource prior to screening, for 39% screening increased their awareness of ways the cancer center supports families and about a quarter believed screening changed their interactions with and resources received from their team.

**Table 4:** Frequencies of patient and caregiver interview codes

Interview themes and codes	Year 1			Year 2			Year 3			Years 1–3	
	PT *n*	CGR *n*	Total %	PT *n*	CGR *n*	Total %	PT *n*	CGR *n*	Total %	*n*	%
*OPSP survey experience*											
Survey was easy to complete/comprehensive	4	18	96%	2	10	80%	1	11	92%	46	90%
Survey was difficult to complete	0	1	4%	0	3	20%	0	1	8%	5	10%
*OPSP survey length*											
Survey length was acceptable	4	14	82%	2	9	63%	1	8	82%	38	79%
Survey length was not acceptable	0	4	18%	0	4	27%	0	2	18%	10	21%
*Education and communication about OPSP*											
Sufficient communication/education	4	14	86%	2	11	93%	1	7	67%	39	83%
Did not have sufficient communication/education	0	3	14%	0	1	7%	0	4	33%	8	17%
*Modes of communicating needs*											
Perceived advantages to communicating needs via survey	1	8	41%	1	3	29%	1	6	58%	20	42%
No difference between survey/sharing directly	1	0	5%	0	2	14%	0	3	25%	6	13%
Preference for direct communication with care team	2	10	55%	1	7	57%	0	2	17%	22	46%
*Impact of OPSP on identifying/communicating needs*											
Helped identify/communicate needs	1	7	38%	2	5	54%	1	8	75%	24	52%
Did not help identify/communicate needs	3	10	62%	0	6	46%	0	3	25%	22	48%
*Impact of OPSP on interactions with care team*											
Facilitated or enhanced care team interactions	0	6	27%	0	2	17%	0	4	33%	12	26%
No change in interactions with care team	4	12	73%	2	8	83%	1	7	67%	34	74%
*Impact of OPSP on family resources*											
Enhanced access to or awareness of resources	0	3	14%	0	5	38%	1	4	45%	13	29%
Did not change resources for family	4	14	86%	2	6	62%	0	6	55%	32	71%
*Impact of OPSP on the cancer center as a resource*											
Strengthened perception of cancer center as a resource	0	5	26%	2	3	36%	1	7	62%	18	39%
Perception remained positive and unchanged	4	10	74%	0	9	64%	0	5	38%	28	61%
*Cancer center responsiveness to family needs/strengths*											
Suggestions to improve support for families	1	6	39%	0	1	10%	1	3	33%	12	30%
Current support adequately meets family needs	3	8	61%	1	8	90%	0	8	67%	28	70%

Frequencies reflect coded responses from *n* = 7 patients (PT) and *n* = 45 caregivers (CGR) across year 1 (2023), year 2 (2024), and year 3 (2025). Percentages were calculated using question-specific denominators (i.e., the number of participants who responded to each item). Because not all participants answered every question, *n* varies by item. “Total” represents aggregated frequencies across years 1–3

*OPSP* oncopsychosocial screening program, *PT* patient, *CGR* caregiver

Responses from oncologists, nurse practitioners, and social workers reflected more positive feedback on implementation strategies (112 coded responses) than barriers (66 coded responses). Identified barriers were impact on workflow (81%), having access to specific screening responses to guide follow-up (8%), uncertainty of value added (8%), and potential burden of survey completion on patients/caregivers (3%). However, clinicians also highlighted effective implementation strategies from initiation through adaptations: the cancer center valuing universal psychosocial screening (35%), timely receipt of screening results (23%), easy-to-access/understandable results (19%), preparation/education on how to review and follow-up on screening results with patients/caregivers (17%), and screening created additional ways to communicate with patients/caregivers about needs and concerns (6%).

### Adaptations and sustainability

Among 55 adaptations, 45 (82%) were made to five components of the screening program: education, administrative workflow, screening assessment and user interface, clinical workflow, and sustainability. *Education* adaptations (9%, *n* = 5) included changes to education/training (e.g., 1:1 education for new clinicians or those unable to attend scheduled sessions). *Screening administrative workflow* (18%, *n* = 8) adaptations focused on operational processes (e.g., adjusted timing for sending surveys to patients/caregivers prior to scheduled appointments). *Screening assessment/user interface* (36%, *n* = 16) adaptations consisted of changes to the screening surveys, interface with families, or timing (e.g., added SSPedi [23] for relevance to clinical care, removed redundant measures of physical quality of life and neurocognition to reduce burden, added open-ended “agenda setting” questions on current concerns). *Clinical workflow* (18%, *n* = 8) adaptations involved changes to clinical processes (e.g., added interventions to social work note template to ease documentation burden, updated score summary reports to provide specific information on screening survey responses). *Sustainability* (18%, *n* = 8) adaptations were made to promote long-term program continuity (e.g., developed a more efficient EHR-based patient eligibility report, built automated alerts to screening team regarding screening initiation/completion, and discontinued in-clinic completion due to limited feasibility across sites and missed same-day results within clinical workflows). Changes were also made to tracking logs, databases, and interviews to improve *quality improvement project workflow* (18%, *n* = 10).

## Discussion

Consistent with the Psychosocial Standards of Care in Childhood Cancer [3, 6], using rigorous quality improvement processes, we achieved development, implementation, and modification of a psychosocial screening program across our large cancer center. Patients, caregivers, and clinicians generally reported that screening was acceptable, providing an additional, important opportunity to share needs and access resources. We also noted that the report of screening results prompted clinicians to discuss needs with patients and caregivers and to collaborate with their colleagues to ensure needs were being met whether or not screening indicated that follow-up was required. However, patients and caregivers highlighted the need for a shorter screening, and challenges occurred in fully integrating screening processes into the EHR. Our quality improvement process of phased implementation and applying REAM [19, 20] allowed for ongoing review and modification of the program over time to address barriers as they were identified, potentially improving our implementation outcomes.

Children’s cancer programs that aim to establish screening programs to meet the standards face barriers to universal screening depending on program characteristics (e.g., size, hospital, and community resources), culture around patient/family assessments, and flexibility of the EHR. Continual review of feedback and real-time adaptations guided by REAM [19, 20] were critical to achieving our aims. Although large in terms of patient population size, complexity, and geography, characteristics of our cancer center (e.g., integrated, multidisciplinary psychosocial services) presented opportunities to identify and address multilevel implementation barriers, sufficient staffing to respond to identified needs, and experience with multidisciplinary partnerships around innovative programming. In addition, our screening team included cancer center leaders who championed the screening program and fostered buy-in to improve access to care. These factors enabled significant progress toward universal screening and helped establish processes that may offer helpful implementation strategies for other children’s cancer programs including those that are less resourced. For example, together, center, hospital, and community resources form the foundation of supports for patients and caregivers during and after treatment, and the Blueprint for Care can be developed uniquely for each childhood cancer program based on the types and range of resources accessible to families. We also believe that screening at key transitions in the cancer trajectory allows for a “check-in” to target limited resources and services to meet those identified with the most acute needs.

Attention to barriers at the patient/caregiver and program/institution levels generated screening implementation strategies generalizable to other children’s cancer programs [12, 14, 36, 37]. The multilevel implementation strategies based on ERIC [33] (language accessibility, education on value of screening for all involved, ensuring communication with patients/caregivers and among the multidisciplinary team about screening results, attention to burden and workflow, screening champions) are consistent with those noted in other studies of psychosocial screening in childhood cancer [12, 14, 37], as well as in a recent systematic review on implementing patient-reported outcomes in adult oncology [36]. Not all barriers were fully addressed, and some barriers (e.g., impact of workflow on patient-caregiver-clinician communication and documentation) represent significant challenges to achieving universal screening. Across implementation phases and with expansion to most patients, the screening team experienced increasing workload for which we tracked time and effort and made adaptations to automate more processes and remove strategies that did not impact care. These decisions involved difficult tradeoffs. For example, we decided to stop screening in the main clinic because this high resource strategy was offered in only one setting and results were received too late to be addressed in the visit; however, this may have reduced access to screening for those with high psychosocial needs.

Psychosocial screening integrated into the EHR remains a goal as does the need to optimize workflow efficiencies to reduce clinical and screening staff demands, influence patient-caregiver-clinician communication about screening results, and support sustainability. Related to EHR integration, the social work team achieved high rates of documented follow-up and multidisciplinary collaboration. In contrast, limited medical clinician documentation indicates that more can be done to leverage the EHR to facilitate screening workflow. Based on our qualitative interviews with oncology providers, this discrepancy in documentation of follow-up did not reflect their recognition of the importance of screening or their willingness to assess and address identified needs. Rather, it was related to the format through which they received results and the infrequency of screening so that the report of results was not easily visible while prepping for clinic visits. All agreed that an EHR prompt would have served as an effective nudge for providers. At our center, we continue to pursue full EHR integration of psychosocial screening facilitated by improved visit scheduling, diagnosis documentation, and treatment end-date tracking in the EHR. While using REDCap [21] allows us to administer screening surveys across timepoints, disease groups, sites, and languages as well as analyze, refine, and optimize the program in real time, retaining all other program components in the EHR (screening invites, results communication, etc.) will allow for the eventual, seamless transition to fully EHR-based screening.

For this screening program, we were able to partner with the CHOP’s Language Services for accurate, reliable translation of measures, instructions, and communications in Spanish and Arabic, and the interpreter team facilitated conversations with families to follow up on screening. Screening in the languages most commonly used in children’s cancer programs in the USA is important given the number of patients/families who speak languages other than English and emerging data regarding health inequities in childhood cancer outcomes [38–40]. Caregivers who speak languages other than English may have difficulties fully understanding medical consent, expressing concerns related to their sociocultural contexts, and accessing the resources available to them [41]. There are significant disparities in cancer care and outcomes for Hispanic children who are less likely to participate in research [42] or receive follow-up care [43], and they are at risk for parental psychological difficulties [44]. Spanish-speaking caregivers in the USA believe their child would have received better cancer care if they spoke English, and language barriers can impact how families engage in care [42].

This report of lessons learned in establishing a psychosocial screening program *may* facilitate implementation of the Psychosocial Standards of Care. We acknowledge that we had resources to rigorously launch, continuously evaluate, and make modifications to implementation strategies. Nevertheless, identified facilitators and strategies that support sustainability, such as convening a multidisciplinary working group, educating patients, caregivers, and oncology providers about the importance of screening, and enhancing efficiencies by reducing measure redundancies and automating screening processes, may be applicable across children’s cancer programs. Ultimately, for children’s cancer programs, findings have the potential to inform the sustainable implementation of comprehensive, evidence-based, screening in multiple languages at key transitions in cancer treatment toward the goal of improved access to and engagement in care.

## Data Availability

The data that support the findings of this study are not openly available due to reasons of sensitivity and are available from the corresponding author upon reasonable request.
